# Mercury Reduces Avian Reproductive Success and Imposes Selection: An Experimental Study with Adult- or Lifetime-Exposure in Zebra Finch

**DOI:** 10.1371/journal.pone.0095674

**Published:** 2014-04-23

**Authors:** Claire W. Varian-Ramos, John P. Swaddle, Daniel A. Cristol

**Affiliations:** 1 Biology Department, Colorado State University – Pueblo, Pueblo, Colorado, United States of America; 2 Institute for Integrative Bird Behavior Studies, Biology Department, The College of William and Mary, Williamsburg, Virginia, United States of America; Oak Ridge National Laboratory, United States of America

## Abstract

Mercury is a global pollutant that biomagnifies in food webs, placing wildlife at risk of reduced reproductive fitness and survival. Songbirds are the most diverse branch of the avian evolutionary tree; many are suffering persistent and serious population declines and we know that songbirds are frequently exposed to mercury pollution. Our objective was to determine the effects of environmentally relevant doses of mercury on reproductive success of songbirds exposed throughout their lives or only as adults. The two modes of exposure simulated philopatric species versus dispersive species, and are particularly relevant because of the heightened mercury-sensitivity of developing nervous systems. We performed a dosing study with dietary methylmercury in a model songbird species, the zebra finch (*Taeniopygia guttata*), at doses from 0.3 – 2.4 parts per million. Birds were exposed to mercury either as adults only or throughout their lives. All doses of mercury reduced reproductive success, with the lowest dose reducing the number of independent offspring produced in one year by 16% and the highest dose, representing approximately half the lethal dose for this species, causing a 50% reduction. While mercury did not affect clutch size or survivorship, it had the most consistent effect on the proportion of chicks that fledged from the nest, regardless of mode of exposure. Among birds exposed as adults, mercury caused a steep increase in the latency to re-nest after loss of a clutch. Birds exposed for their entire lifetimes, which were necessarily the offspring of dosed parents, had up to 50% lower reproductive success than adult-exposed birds at low doses of methylmercury, but increased reproductive success at high doses, suggesting selection for mercury tolerance at the highest level of exposure. Our results indicate that mercury levels in prey items at contaminated sites pose a significant threat to populations of songbirds through reduced reproductive success.

## Introduction

Mercury is a naturally occurring and anthropogenically emitted element that can diminish reproduction and survival in organisms. Human population growth and global climate change will likely exacerbate the problems of mercury pollution due to increases in coal combustion, forest fires, and temperature-dependent biological methylation of inorganic mercury [1]. Once mercury is converted to methylmercury in the environment it readily enters food webs and can biomagnify to toxic concentrations in predatory species, including fish-eating and insectivorous birds [2], [3]. While the lethal effects of mercury on birds and other fish-eating vertebrates have long been known, researchers have more recently uncovered an array of sublethal effects that may have significant fitness consequences on both aquatic and terrestrial birds [4]-[6]. Sublethal mercury accumulation, combined with other stressors such as habitat loss, has been proposed as a serious threat to numerous bird species of high conservation concern (e.g., rusty blackbird, *Euphagus carolinus* [7]; California black rail, *Laterallus jamaicensis* [8]; saltmarsh sparrow, *Ammodramus caudacutus* [9]; California clapper rail, *Rallus longirostris* [10]; Bicknell's thrush, *Catharus bicknelli*, [11]).

While mercury has long been a contaminant of concern for fish-eating birds, recently it has been recognized that terrestrial songbirds are also at risk for mercury contamination [3]. Average blood mercury levels as high as 7 parts per million measured on a wet weight basis (ppm ww) have been found at sites with point source contamination [3]. However, global increases in circulating mercury have resulted in elevated mercury levels in some vulnerable ecosystems which then accumulates in birds living in these ecosystems. In particular, birds living in bogs, estuaries, and other wetlands are at elevated risk, and average blood mercury levels of 0.05 – 0.9 ppm ww have been reported depending on species and season [7], [12], [13]. Birds living in temperate and tropical high elevation forests may have a moderate risk of mercury accumulation with average blood mercury levels of 0.06 – 0.5 ppm ww [11], [14]. Atmospheric mercury has recently been shown to accumulate in feathers of songbirds in the remote southern Appalachian mountain ecosystems to average levels of 0.5 ppm ww [15]. These studies all report total mercury in blood and feathers which are assumed to contain almost entirely methylmercury, however, they do not tell the entire story as some birds have the ability to demethylate mercury [16], [17].

Mercury may impact avian reproduction through disruption of the endocrine system [18], [19], alteration of pairing or parenting behavior [4], [20], [21], and/or direct embryo toxicity [22]. Several field studies have demonstrated reproductive effects of mercury in birds. A study of tree swallows (*Tachycineta bicolor*), a model songbird species for field ecotoxicology studies, found a 20% reduction in the number of offspring produced in a free-living population where the average parental blood mercury was 3.03±0.15 ppm ww [23]. Another insectivorous songbird, the Carolina wren (*Thryothorus ludovicianus*), experienced a 20% decline in probability of a successful nest with each 1.0 ppm increase in blood mercury from 0.0 – 4.0 ppm ww [24]. Several studies in common loons (*Gavia immer*) have indicated a benchmark of reproductive suppression at approximately 2.5 ppm ww in the blood and total reproductive failure at approximately 5 ppm ww [25]. These field studies establish a correlation between mercury and reproductive suppression in birds, but do not establish causation. Therefore, lab based dosing studies are also necessary. A series of dosing studies on captive mallards (*Anas platyrhynchos*), combined with direct injection of mercury into the eggs of numerous avian species, has established that there is considerable inter- and intra-specific variation in sensitivity of reproduction to mercury [22]. Mallards are one of the least sensitive species known, with minimum dietary doses of 0.5 – 4 ppm required to depress offspring viability in different studies of this species [26]. A captive dosing study has also found reproductive effects of methylmercury in American kestrels (*Falco sparverius*) at a dietary dose of 0.7 ppm [27]. However, no dosing study has yet investigated the reproductive effects of mercury in songbirds though we know songbirds are often exposed to mercury contamination [3].

While dosing captive birds is a powerful tool for detecting small effects and establishing causation, meaningful application of the results of dosing studies requires an appreciation of comparable data from the field [28]. The proximate determinant of mercury levels in birds is the mercury concentration in prey items, and the doses used for the present study were designed to span the relevant environmental range for insectivorous terrestrial songbirds. The 0.3 ppm (0.35 ppm measured on a dry weight basis (dw)) dose approximates the upper end of the range for forest-dwelling spiders sampled in remote mountains of the northeastern U.S.A. influenced only by atmospheric deposition (mean value 0.17 ppm dw, [29]), or grassland-dwelling spiders in an industrialized watershed in China (mean value 0.13 ppm dw, [30]), or the average concentration for grasshoppers (0.3 ppm dw) and caterpillars (0.4 ppm dw) in riparian forests and grasslands downstream of a heavily contaminated industrial point source in Virginia, U.S.A. [3]. The 0.6 ppm (0.7 ppm dw) dose is equivalent to the highest concentration found in adult black flies emerging from relatively pristine soft-water streams near Algonquin Park in Canada (range 0.15 – 0.75 ppm dw, [31]) or the average for spiders collected at the forest breeding wetlands of rusty blackbirds in the northeastern U.S.A. and Maritime Canada (∼0.6 ppm dw, [32]). The 1.2 ppm (1.4 ppm dw) dose is similar to the average value for spiders collected in forests and grasslands downstream of a heavily contaminated industrial site in Virginia, U.S.A (1.2 ppm dw, [3]), and is close to samples of terrestrial and aquatic-emergent flying insects eaten by swallows downstream of the same site (0.97 ppm dw, [33]). The highest dose, (2.4 ppm dw) was intended to be slightly beyond the range expected for any but the most extreme wildlife exposures, such as that experienced by predators of large fish.

Because mercury has a long half-life in biological organisms (e.g. 116 days in young loons [34]), and birds are highly mobile, exposure may occur during a variety of life stages. Female birds deposit accumulated mercury into their eggs [35]–[37], so the developing nervous system may be exposed *in ovo* and as a nestling. Birds acquire mercury primarily through locally foraged prey items, so young birds on contaminated sites will get additional exposure until they disperse to establish their own breeding territory. If an entire region is contaminated and the bird species in question is non-migratory, exposure will be life-long. Conversely, birds raised on uncontaminated sites may be exposed to mercury only after they become adults, if they disperse to a contaminated site. Birds that migrate long distances may spend part of each year exposed to mercury, creating even more complex exposure scenarios. The timing of mercury exposure may impact the type and severity of the response in birds. In this study we simulated the first two scenarios: lifetime-exposure in which a non-migratory bird is raised on a contaminated site and continues to be exposed throughout life, and adult-exposure in which a non-migratory bird disperses to a contaminated site and spends its reproductive life exposed to mercury. We performed a dosing study with dietary methylmercury fed to a model songbird species, the zebra finch (*Taeniopygia guttata*), to determine the effects of environmentally relevant doses of mercury on reproductive success of birds exposed throughout their lives or only as adults. Because mercury is thought to have a greater impact during development, we predicted that the birds exposed throughout their lives would be more sensitive to mercury exposure and show reproductive suppression at a lower dose.

## Materials and Methods

### Study species

Zebra finches are the passerine species most commonly used in laboratory studies. Their biology has been very well studied [38] and their genome has recently been sequenced [39] making them an ideal model system. They are a granivorous bird native to Australia. They are very easy to keep in captivity and unlike many other species, will breed continuously when provided adequate resources. This last trait makes them well suited for reproductive studies such as this one as they will complete many breeding attempts in a single year allowing for estimates of lifetime reproductive success in a relatively short period of time.

### Experimental design

All research was conducted at The College of William and Mary aviary in Virginia, USA, between February 2011 and June 2013. This study was carried out in accordance with the recommendations in the Guide of the Care and Use of Laboratory Animals of the National Institutes of Health. All procedures and protocols were approved and overseen by The College of William and Mary's Institutional Animal Care and Use Committee (IACUC 2012-05-23-7982). The birds used for the adult-exposure portion of this study were bred from an existing captive population of zebra finches. All birds were of known parentage, previously unexposed to mercury, sexually mature, and less than 400 days of age at the onset of the study. Birds were maintained indoors under constant environmental conditions (14∶10 light:dark photoperiod, at approximately 22°C), with *ad libitum* access to food, vitamin-enriched water (Vitasol), oyster shell grit, and a cuttlefish bone. Each treatment group was fed a commercial pelletized diet (Zupreem FruitBlend) dosed with 0.0, 0.3, 0.6, 1.2, or 2.4 ppm ww methylmercury cysteine. The lower doses (0.3 and 0.6 ppm) were chosen to reflect the mercury content of common insectivorous songbird prey items reported for numerous habitats including the South River, a contaminated watershed in western Virginia [3]. The higher doses were chosen to represent a worst-case-scenario diet at a contaminated site (1.2 ppm) or to accentuate effects that might be present but difficult to detect at lower mercury levels (2.4 ppm).

### Adult-exposure

180 birds (90 males, 90 females) were randomly assigned to one of the five treatment groups (18 pairs per group). Birds were initially housed in single sex cages and dosed for 10 weeks, at which point blood mercury levels had reached a plateau. After 10 weeks, birds were randomly paired, avoiding inbreeding between any known relatives, and allowed to breed for one year. Birds were maintained on treatment diets for the entire course of the year. We housed birds in pairs with a plastic nest box and *ad libitum* hay for nesting material. Treatment cages were assorted into four experimental rooms with each treatment spread between three rooms and each room containing representatives of at least 3 treatment groups.

Over the course of the year, reproduction was monitored daily. Every egg was marked with a sequential number in permanent marker on the day it was laid. When chicks hatched, the natal down was colored with a non-toxic Crayola© marker to allow for individual identification. Chicks were banded with a uniquely numbered aluminum band at 10 days after hatching. When chicks reached 50 days of age, they were removed from the parental cage and maintained in flocks on the same diet for use in the second part of the study (below). This daily monitoring allowed us to determine the fate of every egg from laying to independence, producing very accurate and complete measures of reproductive success for each breeding pair. The first clutch produced by every pair was removed to determine egg mercury concentration and standardize conditions for measuring the number of days until a new nest was initiated (inter-clutch interval). Blood mercury levels were measured monthly in each bird. A small (approximately 30 µL) blood sample was taken from the brachial vein by puncturing it with a 30-gauge sterile needle. The blood droplet was collected in a heparinized microcapillary tube and frozen at −20 °C until mercury analysis (below).

### Lifetime-exposure

Birds for the lifetime-exposure portion of the study were the offspring of the adult-exposed birds, and were also sexually mature and less than 400 days of age at the onset of reproduction. Because not all pairs from the adult-exposure portion of the experiment reproduced successfully, not all pairs were represented by offspring in the second portion of the study. For those initial pairs that did produce young, one randomly selected male and female offspring was chosen from each pair to go into the lifetime-exposure portion of the study. To bring the number up to 18 pairs per treatment group, additional siblings were randomly chosen with no more than 2 males and 2 females from any one pair from the adult-exposure portion of the study. Males and females were randomly paired within treatment groups, avoiding any inbreeding between known relatives. Birds were housed and reproduction monitored as described above for the adult-exposure portion of the study.

### Food Preparation

The food was dosed with an aqueous solution of methylmercury cysteine, which is thought to be the form of mercury found in a natural avian diet [40]. Methylmercury cysteine was made by dissolving methylmercury chloride in 100% ethanol and combining in a 1∶99 ratio with degassed deionized water containing a 2 X molar excess of cysteine to create a 40 ppm stock solution. Food was then prepared by diluting the stock solution to the desired concentration and mixing in with food at a 1∶9 ratio by weight. Food was then homogenized in a rock tumbler for 30 minutes. Control food was prepared by mixing a solution of water and cysteine with the food. Each batch of food was tested to confirm that it fell within 10% of the target concentration.

### Mercury analysis

Total mercury concentrations for blood and food were analyzed using a DMA-80 (Direct Mercury Analyzer, Milestone Scientific). All samples were run fresh (i.e. not freeze-dried). We followed standard quality control procedures for all analyses. The DMA-80 was calibrated approximately every two months or as needed throughout the study. Certified standard reference materials (National Research Council Canada) and machine and sample blanks were run with every batch of 20 samples to check for calibration stability and contamination. The recovery of standard reference materials over the entire two years of the study was within acceptable limits (DORM-3: 103.0±0.1%, n = 1814; DORM-4: 101.7±0.4%, n = 255; DOLT-3: 99.7±0.4%, n = 66; DOLT-4: 100.7±0.1%, n = 1954). When reference material was spiked into bird blood the recovery was 98.8±0.7%, n = 26. Duplicate blood samples were included with approximately every 20 blood samples as available and the relative percent difference was 7.5±1.4%, n = 45. The average calculated minimum detection limit was 0.008±0.001 ppm.

### Statistical methods

We used five measures of reproductive performance: the total number of independent (50-day old) offspring produced in one year (independent offspring); median number of eggs in all clutches produced in one year where a clutch is defined as a group of eggs laid on sequential days and separated by at least 4 days on which no egg was laid (clutch size); proportion of eggs laid that hatched (hatching success); proportion of chicks hatched that survived to leave the nest box (fledging success); and the number of days between when the first clutch produced was removed and the first new egg of the next clutch was laid (latency to re-nest). We also analyzed adult mortality rates by recording whether both members of the pair survived the entire year of the study (survival). We consider the number of independent offspring to be a measure of lifetime reproductive success (or fitness) as it takes into account all components of reproduction as well as pair survival. Because captive zebra finches maintained on long day photoperiods attempt to breed continuously, we interpreted one year of consecutive breeding as being equivalent to the lifetime reproductive success of a small songbird breeding over several years.

All statistics were performed using SPSS 19 (IBM). We used generalized linear mixed models for all analyses of reproductive measures. For each, treatment level and type of mercury exposure (adult or lifetime) were used as fixed effects. Room was included as a random effect with a scaled identity covariance matrix to control for any differences in environment between rooms. All count measures (independent offspring, clutch size, and latency to re-nest) were modeled using a Poisson distribution and a log link function. Proportion measures (hatching success, fledging success) were modeled using a binomial distribution and a logit link. Survival was designated as a binary (1 = both members of the pair survived for 1 year, 0 =  at least one member of the pair died within the year) and modeled using a binomial distribution and a logit link. We conducted post hoc comparisons of all treatments to the control using a sequential Bonferroni adjustment of the *p* value and interpreted two-tailed tests of significance. All means are presented with standard errors throughout.

## Results

### Blood mercury levels

**Figure 1:**
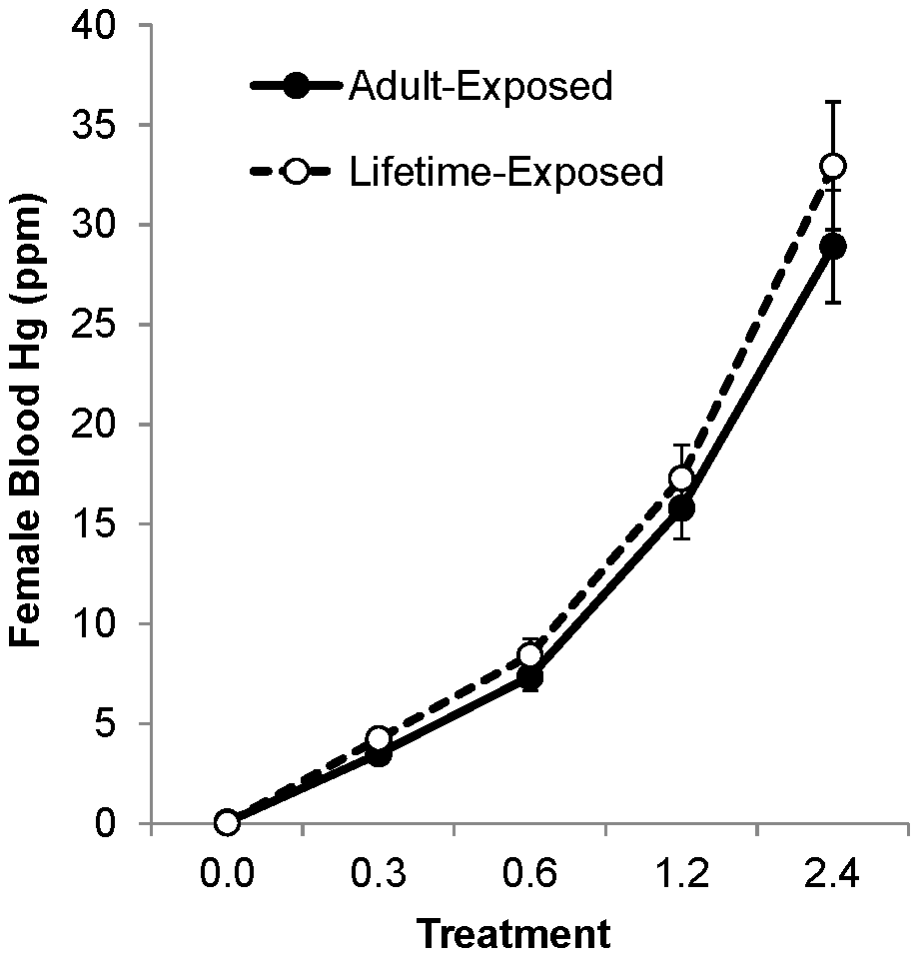
Average blood mercury values for each dietary dose of adult-exposed and lifetime-exposed zebra finches. Adult-exposed averages are represented by filled circles and solid lines; lifetime-exposed averages are represented by hollow circles and dashed lines. Values are means and bars are one S.E.

Dietary mercury dosing effectively raised blood mercury levels with each doubling in dietary dose corresponding to an approximate doubling in blood mercury levels (Fig. 1). On average the blood mercury level of the birds was 13.2±0.2 times the dietary dose. For all doses, the lifetime-exposed birds had slightly, but not significantly, higher blood mercury concentrations than the adult-exposed birds (Wald χ^2^ = 0.57, df = 1, P = 0.45; Fig. 1)

### General effects of mercury exposure

**Figure 2:**
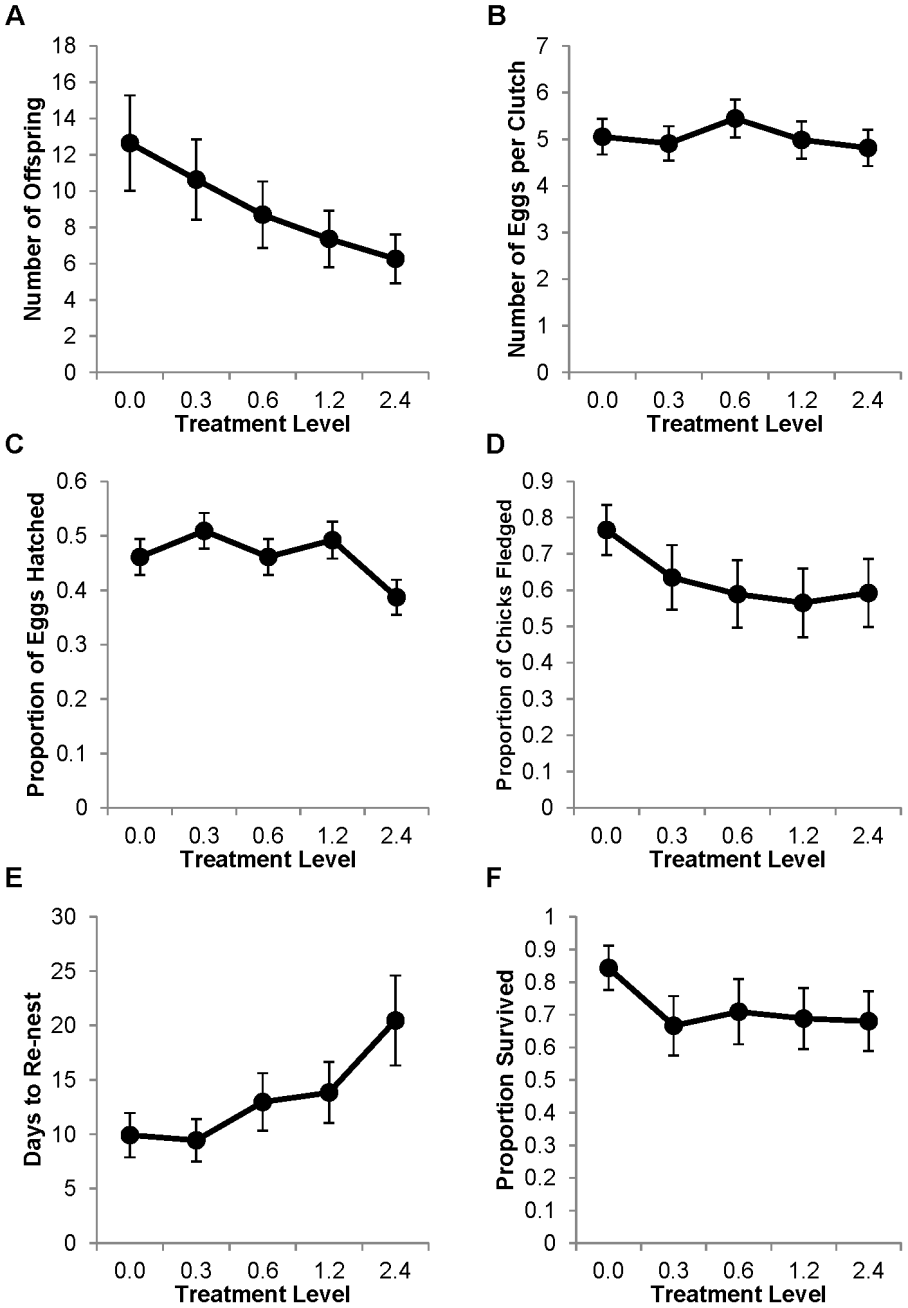
Effects of dietary mercury on zebra finch reproduction. All points are model averages from the generalized linear mixed models. Bars are one S.E. A) The average total number of independent offspring produced per pair in one year of reproduction. B) The average clutch size. C) The proportion of eggs laid that hatched. D) The proportion of hatched chicks that survived to leave the nest. E) The number of days between removal of the first clutch of eggs and laying of the second clutch. F) The probability that both members of the pair survived for one year.

Mercury markedly decreased reproductive success as measured by the number of independent offspring produced in one year (Fig. 2A; F_4,170_ = 23.52, P<0.001). On average, pairs produced approximately seven broods during the year of the study. Post hoc comparisons revealed that all treatments produced significantly fewer offspring than the control, such that the 0.3 ppm treatment level produced a 16% reduction in reproductive success (P = 0.03), the 0.6 ppm treatment level produced a 31% reduction (P = 0.001), the 1.2 ppm treatment level produced a 42% reduction (P<0.001), and the 2.4 ppm treatment level produced a 50% reduction (P<0.001).

To help understand which components of fitness were affected by mercury exposure, we further analyzed how mercury influenced fledging success, latency to re-nest (i.e. an estimate of inter-clutch interval), hatching success, clutch size, and adult survival (Table 1). Fledging success was significantly reduced by mercury (Fig. 2D; F_4,150_ = 16.63, P<0.001) with all treatment levels having significantly lower fledging success relative to the control (P<0.001). Latency to re-nest was also impacted by mercury (Fig. 2E; F_4,155_ = 39.45, P<0.001) but only treatment levels of 0.6 ppm and above required a statistically significantly greater number of days to re-nest relative to the control (P<0.02, in all cases). To reiterate, there was no significant difference between the 0.3 ppm treatment and the control in the number of days to re-nest (P = 0.55). There was a significant impact of mercury treatment on hatching success (Fig. 2C; F_4,155_ = 10.10, P<0.001). The 0.3 ppm treatment level had slightly higher hatching success than the control (P = 0.03) while the 2.4 ppm treatment level had notably lower hatching success than the control (P = 0.001). Mercury treatment had no detectable effects on either clutch size (Fig. 2B; F_4,158_ = 0.38, P = 0.82) or adult survival (Fig. 2F; F_4,170_ = 0.82, P = 0.51).

**Table 1:** Results of Generalized Linear Mixed Models.

Analysis	Factor	F Stat.	DF	P
Independent Offspring	Model	16.69	9, 170	< 0.001
	Mercury Level	23.52	4, 170	< 0.001
	Type of Exposure	3.22	1, 170	0.08
	Level * Type	15.01	4, 170	< 0.001
Clutch Size	Model	0.28	9, 158	0.98
	Mercury Level	0.38	4, 158	0.82
	Type of Exposure	0.59	1, 158	0.44
	Level * Type	0.08	4, 158	0.99
Hatching Success	Model	28.58	9, 155	< 0.001
	Mercury Level	10.10	4, 155	< 0.001
	Type of Exposure	79.47	1, 155	< 0.001
	Level * Type	38.72	4, 155	< 0.001
Fledging Success	Model	11.39	9, 150	< 0.001
	Mercury Level	16.63	4, 150	< 0.001
	Type of Exposure	19.04	1, 150	< 0.001
	Level * Type	4.74	4, 150	< 0.01
Latency to Re-nest	Model	59.59	9, 155	< 0.001
	Mercury Level	39.45	4, 155	< 0.001
	Type of Exposure	5.196	1, 155	0.02
	Level * Type	67.73	4, 155	< 0.001
Adult Survival	Model	2.03	9, 170	0.04
	Mercury Level	0.82	4, 170	0.51
	Type of Exposure	11.74	1, 170	< 0.01
	Level * Type	0.69	4, 170	0.60

### Effects of lifetime vs. adult exposure

**Figure 3:**
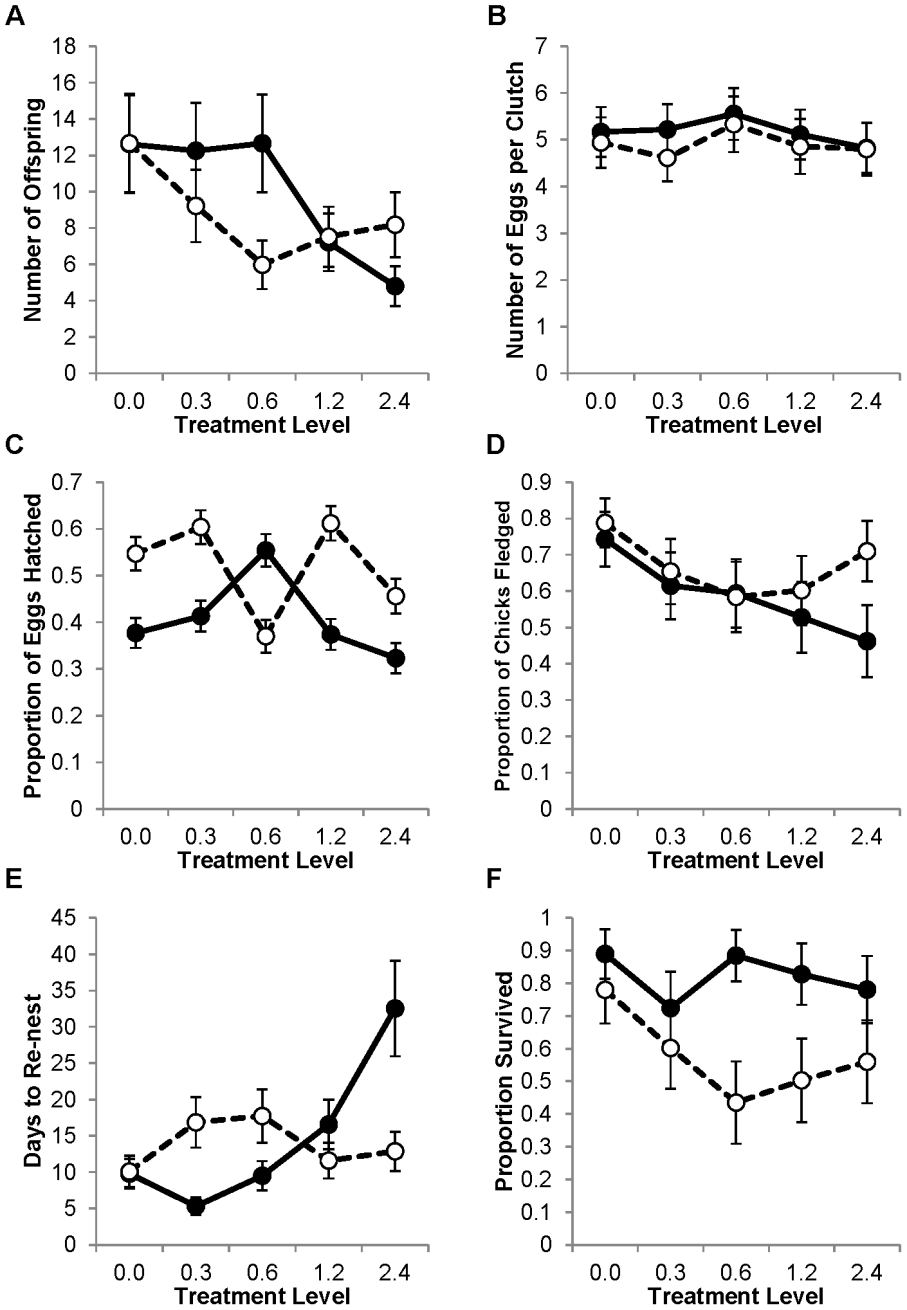
Differences in the effects of mercury on reproduction between adult-exposed and lifetime-exposed zebra finches. Adult-exposed averages are represented by filled circles and solid lines; lifetime-exposed averages are represented by hollow circles and dashed lines. All points are model averages from the generalized linear mixed models. Bars are one S.E. A) The average total number of independent offspring produced per pair in one year of reproduction. B) The average clutch size. C) The proportion of eggs laid that hatched. D) The proportion of hatched chicks that survived to leave the nest. E) The number of days between removal of the first clutch of eggs and laying of the second clutch. F) The probability that both members of the pair survived for one year.

The type of mercury exposure (adult vs. lifetime) had an impact on how mercury affected reproductive success (Fig. 3A; F_4,170_ = 15.01, P<0.001). In the lower mercury exposure treatments, specifically the 0.3 and 0.6 treatments, lifetime-exposed birds had significantly lower total reproductive success, as measured by number of independent offspring, than adult-exposed birds (P = 0.02 and <0.001, respectively). This is consistent with lifetime-exposed birds being more sensitive to the detrimental effects of mercury exposure. In striking contrast, in the highest mercury exposure treatment (i.e. 2.4 ppm) lifetime-exposed birds had higher reproductive success than adult-exposed birds (P<0.01). As lifetime-exposed birds were the offspring of adult-exposed birds, this observation is consistent with a rapid, evolved response to artificial selection for mercury tolerance at the highest level of mercury exposure.

As with the previous analyses that grouped all birds together, we dissected components of breeding and survival to help explain fitness differences between lifetime- and adult-exposed birds. There was an interaction between the effects of type of mercury exposure and mercury level on fledging success (Fig. 3D; F_4,150_ = 4.742, P = 0.001). Birds with lifetime-exposure to 2.4 ppm had higher fledging success than those exposed to 2.4 ppm only as adults (P<0.001). Latency to re-nest also revealed a significant interaction between type of exposure and treatment level (Fig. 3E; F_4,155_ = 67.73, P<0.001), with lifetime-exposed birds being affected more than adult-exposed birds at lower dose treatments (0.3 and 0.6 ppm, P<0.001) but being less impacted at higher doses (1.2 and 2.4 ppm, P<0.001). There was a significant interaction between exposure type and treatment in hatching success as well (Fig. 3C; F_4,155_ = 38.72, P<0.001), but the pattern is biologically non-intuitive. There was no significant interaction between exposure type and treatment level in either clutch size (Fig. 3B; F_4,158_ = 0.081, P  = 0.99) or adult survival (Fig. 3F; F_4,170_ = 0.693, P = 0.60), although survival was generally lower in the lifetime-exposed birds (F_1,170_ = 11.744, P = 0.001).

## Discussion

The dietary dosing successfully manipulated blood mercury levels in the different treatments with the lowest two doses (0.3 and 0.6 ppm) accumulating blood mercury levels (approximately 4 and 8 ppm respectively) similar to blood mercury levels seen in wild bird populations in areas with point source contamination [3]. Mercury levels impacted reproductive success, but not all reproductive endpoints (e.g. clutch size) were affected by treatment level (Table 1). The timing of exposure had a significant effect on reproductive success, with developmentally exposed birds showing increased sensitivity to mercury at the lower doses (0.3 and 0.6 ppm) but decreased sensitivity in the highest dose (2.4 ppm). This suggests that there may have been selection for resistance to mercury after only one generation.

### Effect of mercury on reproductive success

Methylmercury exposure reduced reproductive success at all dosing levels in this study. Ecologically, this observation of both adult- and lifetime-exposed birds combined (Fig. 1) approximates the effects of mercury contamination on a free-living population of non-migratory songbirds that contains a mixture of philopatric and dispersive individuals. The percent reduction of independent offspring produced at the lowest dose was similar to what was observed in tree swallows at an industrially-contaminated site (16% reduction at approximately 4 ppm in zebra finch versus 20% reduction at approximately 3 ppm in tree swallow [23]). In contrast, data from Carolina wrens at the same site predicted 80% nest failure at comparable blood mercury levels [24]. The average blood mercury levels of both tree swallows and Carolina wrens at the contaminated South River were approximately 3 ppm, indicating that this is a representative value for passerines at a contaminated site [23], [24]. The reduction in fitness we observed in zebra finches started at our lowest dose of mercury, indicating that zebra finch reproduction, like that of Carolina wrens, is likely impacted by dietary mercury at a level below 0.3 ppm.

### Relative sensitivity

Often in toxicology it is assumed that all members of a particular taxon (e.g. birds) will react similarly to a contaminant, but this may often not be the case. In previous dosing studies, reproductive impairment began at 0.5 ppm dietary mercury in mallards [26] and 0.7 ppm dietary mercury in kestrels [27], suggesting that zebra finches may be somewhat more sensitive to mercury than waterfowl or raptors. White ibis showed a 35% decrease in reproductive success at 0.3 ppm dietary mercury [4]. A study in which eggs of 26 species were injected with mercury concluded that two species of songbirds were more sensitive to mercury than mallards but less sensitive than kestrels or ibis [22]. Our results suggest that dosed zebra finches may, in fact, be no less sensitive than kestrels to reproductive disruption by mercury. Dietary doses of 5 ppm have been shown to cause significant mortality in zebra finches within only 80 days [41] suggesting that this may be close to the lethal dose for chronic exposure. Thus, we found 50% reduction in reproduction at approximately half the lethal dose and 16% reduction at only 6% of the lethal dose. We suggest that these percentages might be useful guideposts in future studies when trying to estimate population injury—zebra finches experienced a substantial loss (∼15%) of reproductive productivity at an exposure level that was only a few percent (∼5%) of the lethal concentration.

### Variation in sensitivity of endpoints

The components of reproductive success that appeared to be most affected by mercury in the zebra finch were fledging success and latency to re-nest. These endpoints are likely influenced by parental behavior, and thus may be more impacted by mercury as it is a known neurotoxin. In the wild, impacts on fledging success may be exacerbated as parents usually have limited food resources and face higher risks of predation. Further study is needed to determine whether the effects on fledging success were due to impacts on nestling physiology or development or whether changes in parental behavior accounted for the difference in nestling survival. Increased length of time between nesting attempts can have a large effect on individual fitness in the field as it can limit an individual's ability to double brood, or prevent re-nesting if a brood is lost to predation. We did not find a strong effect of mercury on hatching success, which is contrary to the prevailing belief that the developing embryo is the most sensitive life stage to mercury [22]. However, the mortality we observed during the nestling period may have been due to the delayed effects of *in ovo* exposure. The interesting pattern we observed in which the 0.3 ppm treatment showed slightly higher hatching success and the 2.4 ppm treatment had much lower hatching success could be an example of hormesis, similar to that recently reported for hatching success in mallards [42].

### Effects of timing of exposure

We also found some important differences between birds with lifetime-exposure to mercury and those exposed only as adults. The lifetime-exposed birds were more sensitive to mercury, showing significant reproductive suppression at the lower doses (0.3 and 0.6 ppm) while the adult-exposed birds did not show reproductive suppression until 1.2 ppm. This could be because there is a greater impact of mercury when exposure occurs during development. Alternatively, the difference could be attributed to a longer period of chronic exposure for the lifetime-exposed birds. Further research that distinguishes early exposure from longer exposure will be necessary to separate these two hypotheses. Our lab is currently investigating this question. Mercury [43], as well as other environmental contaminants [44], [45], is known to have greater effects on developing organisms and these effects may continue throughout the life of the individual [46].

Another difference between the lifetime-exposed birds and the adult-exposed birds occurred at the highest treatment level (2.4 ppm). The lifetime-exposed birds had higher reproductive success than the adult-exposed birds at that dietary concentration. Because the lifetime-exposed birds were the offspring of the adult-exposed birds, this pattern is consistent with rapid adaptation to mercury exposure. Selection pressure was exerted both by the fact that unsuccessful pairs were not represented by offspring in the lifetime-exposed portion of the experiment and through mortality during the nestling stage, as all individuals included in the lifetime-exposure portion had by definition survived exposure as nestlings. In a previous study we found that families of related zebra finches responded differently to mercury exposure, with some genetic families showing little response to mercury contamination [47]. We also know that there is a significant heritable genetic component to blood mercury level accumulation in our population of zebra finches [48]. Therefore, we have evidence that there is a notable genetic component to responses to mercury in this population upon which selection could act.

### Implications for free-living populations

Blood mercury levels produced in this study by the lower doses (approximately 4 ppm for the 0.3 ppm dose and approximately 8 ppm for the 0.6 ppm dose) resulted in a decline in reproductive success similar to one songbird species on industrially contaminated sites (tree swallow, 3 ppm in blood [23]) but considerably less than another (Carolina wren, 3 ppm in blood [24]). These differences could be attributed to the interspecific variation in sensitivity observed among bird species [22]. Additionally, the decreased sensitivity to mercury observed in zebra finches relative to Carolina wrens could be a result of less stressful living conditions within the aviary relative to the field. In captivity, the birds have unlimited access to food and water with no risk of predation. It may be that these stressors (i.e. limited food and predation risk) exacerbate the effects of mercury on free-living birds. Thus the fitness reduction shown here may be an underestimate of the actual harm to wild populations and much caution should be used in applying it to wild birds. However, if our results here were an accurate representation of the effects in the field, the reduction in reproductive success even at the lowest dosing level could potentially lead to population declines, particularly in small or isolated populations. This lends support to the idea that mercury contamination may be of significant conservation concern to populations of birds with high exposure or additional threats. We also feel that our metric of observing a significant loss in productivity at approximately 6% of the lethal mercury exposure level could be used as a conservative starting point for assessing potential population damages. Although this is likely an underestimate (for the reasons explained above), if field workers have information about lethal dietary levels in free-living birds, the results of our study can be used as a starting point for extrapolating expected reductions in reproductive success at lower dietary concentrations.

Another important consideration for applying these findings to free-living birds is that lifetime-exposed birds are more sensitive to mercury contamination than those exposed only as adults. The lifetime-exposed birds can be compared to relatively sedentary or philopatric species that spend their entire lives on contaminated sites or return to their natal area to breed, whereas the adult-exposed birds may be more representative of birds with longer natal dispersal distances that hatch on an uncontaminated site and then immigrate to a contaminated area to breed. Natal dispersal distances vary between species, so even philopatric species may have dispersal distances large enough for them to escape contamination as adults. However, many species of conservation concern are limited by available habitat and are therefore more philopatric than generalist species. Because of this, these already-vulnerable species may be at even greater risk from mercury pollution. In fact, the lifetime-exposed birds in this study had a 27% reduction in reproductive success at the lowest dietary dose used here (0.3 ppm), suggesting that mercury can be a very serious threat to philopatric populations.

We also found evidence suggestive of adaptation to mercury contamination after just one generation of strong selection. If genetic variation for resistance to mercury pollution exists in wild populations as well, which seems reasonable as there was variation in our relatively small zebra finch population, then evolution may occur in philopatric populations relatively rapidly. Genetic variance for tolerance of environmental pollutants has been documented in several invertebrate species [49], [50] and in fish [51]. Similar variation in birds could lead to genetic differences between populations on contaminated and uncontaminated sites, resulting in underestimates of the effects of mercury when birds from contaminated sites are compared to those from reference sites. Additionally, we have previously found that the families that are more resistant to the effects of mercury had lower fitness on control diets [25]. Thus, those birds that are best able to breed on a contaminated site may produce offspring that have lower fitness if they disperse to uncontaminated areas, resulting in another more cryptic cost of mercury on species as a whole.

## References

[1] Hooper MJ, Ankley GT, Cristol DA, Maryoung LA, Noyes PD, et al (2012) Interactions between chemical and climate stressors: A role for mechanistic toxicology in assessing climate change risks. Environ Toxicol Chem 32: 32–48. 10.1002/etc.2043 PMC3601417 23136056

[2] Scheulhammer AM, Meyer MW, Sandheinrich MB, Murray MW (2007) Effects of environmental methylmercury on the health of wild birds, mammals, and fish. Ambio 36: 12–18. 17408187 10.1579/0044-7447(2007)36[12:eoemot]2.0.co;2

[3] Cristol DA, Brasso RL, Condon AM, Fovargue RE, Friedman SL, et al (2008) The movement of aquatic mercury through terrestrial food webs. Science 320: 335. 18420925 10.1126/science.1154082

[4] Frederick P, Jayasena N (2010) Altered pairing behaviour and reproductive success in white ibises exposed to environmentally relevant concentrations of methylmercury. Proc Roy Soc Lond B 278: 1853–1857. 10.1098/rspb.2010.2189 PMC3097836 21123262

[5] Bouland AJ, White AE, Lonabaugh KP, Varian-Ramos CW, Cristol DA (2012) Female-biased offspring sex ratios in birds at a mercury-contaminated river. J Avian Biol 43: 244–251.

[6] Lewis CA, Cristol DA, Swaddle JP, Varian-Ramos CW, Zwollo P (2013) Decreased immune response in zebra finches exposed to sublethal doses of mercury. Arch Environ Contam Toxicol 64: 327–336. 23229191 10.1007/s00244-012-9830-z

[7] Edmonds ST, Evers DC, Cristol DA, Mettke-Hofmann C, Powell LL, et al (2010) Geographic and seasonal variation in mercury exposure of the declining rusty blackbird. Condor 112: 789–799.

[8] Tsao DC, Miles AK, Takekawa JY, Woo I (2007) Potential effects of mercury on threatened California black rails. Arch. Environ. Contam. Toxicol. 56: 292–301. 10.1007/s00244-008-9188-4 18648717

[9] Cristol DA, Smith FM, Varian-Ramos CW, Watts BD (2011) Mercury levels of Nelson's and saltmarsh sparrows at wintering grounds in Virginia, USA. Ecotoxicology 20: 1773–1779. 21698442 10.1007/s10646-011-0710-5

[10] Ackerman JT, Overton CT, Casazza ML, Takekawa JY, Eagles-Smith CA, et al (2012) Does mercury contamination reduce body condition of endangered California clapper rails? Environ Pollut 162: 439–448. 22243896 10.1016/j.envpol.2011.12.004

[11] Rimmer CC, Mcfarland KP, Evers DC, Miller EK, Aubry Y, et al (2005) Mercury concentrations in Bicknell's thrush and other insectivorous passerines in montane forests of northeastern North America. Environ Pollut 162: 439–48. 10.1007/s10646-004-6270-1 15931968

[12] Strom SM, Brady RS (2011) Mercury in swamp sparrows (*Melospiza georgiana*) from wetland habitats in Wisconsin. Ecotoxicology 20: 1694–1700. 21755351 10.1007/s10646-011-0734-x

[13] Winder VL, Emslie SD (2012) Mercury in non-breeding sparrows of North Carolina salt marshes. Ecotoxicology 21: 325–335. 21947667 10.1007/s10646-011-0794-y

[14] Townsend JM, Rimmer CC, Driscoll CT, McFarland KP, Inigo-Elias E (2013) Mercury concentrations in tropical resident and migrant songbirds on Hispaniola. Ecotoxicology 22: 86–93. 23076839 10.1007/s10646-012-1005-1

[15] Keller RH, Xie L, Buchwalter DB, Franzreb KE, Simons TR (2014) Mercury bioaccumulation in Southern Appalachian birds, assessed through feather concentrations. Ecotoxicology 23: 304–316. 24420618 10.1007/s10646-013-1174-6

[16] Kim EY, Murakami T, Saeki K, Tatsukawa R (1996) Mercury levels and its chemical form in tissues and organs of seabirds. Arch Environ Contam Toxicol 30: 259–266.

[17] Eagle-Smith CA, Ackerman JT, Yee J, Adelsbach TL (2009) Mercury demethylation in waterbird livers: does-response thresholds and differences among species. Environ Toxicol Chem 28: 568–577. 18937537 10.1897/08-245.1

[18] Jayasena N, Frederick PC, Larkin ILV (2011) Endocrine disruption in white ibises (*Eudocimus albus*) caused by exposure to environmentally relevant levels of methylmercury. Aquat Toxicol 105: 321–327. 21801696 10.1016/j.aquatox.2011.07.003

[19] Heath JA, Frederick PC (2005) Relationships among mercury concentrations, hormones, and nesting effort of white ibises (*Eudocimus albus*) in the Florida everglades. Auk 122: 255–267.

[20] Hallinger KK, Zabransky DJ, Kazmer KA, Cristol DA (2010) Song differs between birds on mercury-polluted and reference sites. Auk 127: 156–161.

[21] Evers DC, Savoy LJ, DeSorbo CR, Yates DE, Hanson W, et al (2008) Adverse effects from environmental mercury loads on breeding common loons. Ecotoxicology 17: 69–81. 17909967 10.1007/s10646-007-0168-7

[22] Heinz GH, Hoffman DJ, Klimstra JD, Stebbins KR, Kondrad SL, et al (2009) Species differences in sensitivity of avian embryos to methylmercury. Arch Environ Contam Toxicol 56: 129–138. 18421496 10.1007/s00244-008-9160-3

[23] Hallinger KK, Cristol DA (2011) The role of weather in mediating the effect of mercury exposure on reproductive success of tree swallows. Ecotoxicology 20: 1368–1377. 21553259 10.1007/s10646-011-0694-1

[24] Jackson AK, Evers DC, Etterson MA, Condon AM, Folsom SB, et al (2011) Mercury exposure affects the reproductive success of a free-living terrestrial songbird, the Carolina wren (*Thryothorus ludovicianus*). Auk 128: 759–769.

[25] Depew DC, Basu N, Burgess NM, Campbell LM, Ever DC, et al (2012) Derivation of screening benckmarks for dietary methylmercury exposure in the common loon (*Gavia immer*): Rational for use in ecological risk assessment. Environ Toxicol Chem 31: 2399–2407. 22865698 10.1002/etc.1971

[26] Heinz GH, Hoffman DJ, Klimstra JD, Stebbins KR (2010) Reproduction in mallards exposed to dietary concentrations of methylmercury. Ecotoxicology 19: 977–982. 20232247 10.1007/s10646-010-0479-y

[27] Albers PH, Koterba MT, Rossmann R, Link WA, French JB, et al (2007) Effects of methylmercury on reproduction in American kestrels. Environ Toxicol Chem 26: 1956–1866. 10.1897/06-592R.1 17702546

[28] Burger J, Gochfeld M (1997) Risk, mercury levels, and birds: relating adverse laboratory effects to field biomonitoring. Environ Res 75: 160–172. 9417847 10.1006/enrs.1997.3778

[29] Rimmer CC, Miller EK, McFarland KP, Taylor RJ (2010) Mercury bioaccumulation and trophic transfer in the terrestrial food web of a montane forest. Ecotoxicology 19: 697–709. 19960247 10.1007/s10646-009-0443-x

[30] Zhang Z, Wag Q, Zheng D, Zheng N, Lu X (2010) Mercury distribution and bioaccumulation up the soil-plant-grasshopper-spider food chain in Huludao City, China. J Environ Sci 22: 1179–1183. 10.1016/s1001-0742(09)60235-7 21179955

[31] Harding KM, Gowland JA, Dillon PJ (2006) Mercury concentration in black flies Simulium spp. (Diptera, Simuliidae) from soft-water streams in Ontario, Canada. Environ Pollut 143: 529–535. 16490293 10.1016/j.envpol.2005.11.040

[32] Edmonds ST, O’Driscoll NJ, Hillier NK, Atwood JL, Evers DC (2012) Factors regulating the bioavailability of methylmercury to breeding rusty blackbirds in northeastern wetlands. Environ Pollut 171: 148–154. 22922392 10.1016/j.envpol.2012.07.044

[33] Brasso RL, Cristol DA (2008) Effects of mercury exposure on the reproductive success of tree swallows (Tachycineta bicolor). Ecotoxicology 17: 133–141. 17701345 10.1007/s10646-007-0163-z

[34] Fournier F, Karasov WK, Kenow KP, Meyer MW, Hines RK (2002) The oral bioavailability and toxicokinetics of methylmercury in common loon (*Gavia immer*) chicks. Comp Biochem Physiol Part A Mol Integr Physiol 133: 703–714. 10.1016/s1095-6433(02)00140-x 12443928

[35] Evers DC, Taylor KM, Major A, Taylor RJ, Poppenga RH, et al (2003) Common loon eggs as indicators of methylmercury availability in North America. Ecotoxicology 12: 69–81. 12739858 10.1023/a:1022593030009

[36] Henry CJ, Hill EF, Hoffman DJ, Spalding MG, Grove RA (2002) Nineteenth century mercury: Hazard to wading birds and cormorants of the Carson River, Nevada. Ecotoxicology 11: 213–231. 12211695 10.1023/a:1016327602656

[37] Evers DC, Burgess NM, Champoux L, Hoskins B, Major A, et al (2005) Patterns and interpretation of mercury exposure in freshwater avian communities in northeastern North America. Ecotoxicology 14: 193–221. 15931967 10.1007/s10646-004-6269-7

[38] Zann RA (1996) The Zebra Finch: A Synthesis of Field and Laboratory Studies. Oxford University Press.

[39] Warren WC, Clayton DF, Ellegren H, Arnold AP, Hillier LW, et al (2010) The genome of a songbird. Nature 464: 757–762. 20360741 10.1038/nature08819 PMC3187626

[40] Harris HH, Pickering IJ, George GN (2003) The chemical form of mercury in fish. Science 301: 1203. 12947190 10.1126/science.1085941

[41] Scheuhammer AM (1988) Chronic dietary toxicity of methylmercury in the zebra finch, *Poephila guttata* . Bull Environ Contam Toxicology 40: 123–130. 10.1007/BF01689398 3345357

[42] Heinz GH, Hoffman DJ, Klimstra JD, Stebbins KR, Kondrad SL, et al (2012) Hormesis associated with a low dose of methylmercury injected into mallard eggs. Arch Environ Contam Toxicol 62: 141–144. 21604054 10.1007/s00244-011-9680-0

[43] Choi AL, Grandjean P (2008) Methylmercury exposure and health effects in humans. Environ Chem 5: 112–120.

[44] Perera F, Herbstman J (2011) Prenatal environmental exposures, epigenetics, and disease. Reprod Toxicol 31: 363–373. 21256208 10.1016/j.reprotox.2010.12.055 PMC3171169

[45] Caserta D, Graziano A, Lo Monte G, Bordi G, Moscarini M (2013) Heavy metals and placental fetal-maternal barrier: a mini-review on the major concerns. Eur Rev Med Pharmacol Sci 17: 2198–2206. 23893187

[46] Rice DC (1996) Evidence for delayed neurotoxicity produced by methylmercury. Neurotoxicology 17: 583–596. 9086479

[47] Varian-Ramos CW, Swaddle JP, Cristol DA (2013) Familial differences in the effects of mercury on reproduction in zebra finches. Environ Pollut 183: 316–323. 10.1016/j.envpol.2013.07.044 23973883

[48] Buck KA (2013) Evaluating the potential for adaptive response to mercury in captive-dosed zebra finches. MS thesis, The College of William & Mary, Williamsburg, VA, USA.

[49] Lange BW, Langley CH, Stephan W (1990) Molecular evolution of *Drosophila* metallothionein genes. Genetics 126: 921–932. 1981765 10.1093/genetics/126.4.921 PMC1204289

[50] Janssens TKS, Roelofs D, Van Straalen NM (2009) Molecular mechanisms of heavy metal tolerance and evolution in invertebrates. Insect Sci 16: 3–18.

[51] Reitzel AM, Karchner SI, Franks DG, Evans BR, Nacci D, et al (2013) Genetic variation at aryl hydrocarbon receptor (AHR) loci in populations of Atlantic killifish (*Fundulus heteroclitus*) inhabiting polluted and reference habitats. BMC Evol Biol 14: 1–35. 10.1186/1471-2148-14-6 PMC3899389 24422594

